# Flow cytometric analysis of *Xenopus laevis* and *X. tropicalis* blood cells using acridine orange

**DOI:** 10.1038/s41598-018-34631-0

**Published:** 2018-11-02

**Authors:** Kei Sato, Azusa Uehara, Sayaka Kinoshita, Ikki Nomura, Minami Yagi, Yuta Tanizaki, Yu Matsuda-shoji, Atsushi Matsubayashi, Nobuyasu Endo, Yutaka Nagai, Takashi Kato

**Affiliations:** 1 Faculty of Education and Integrated Arts and Sciences, Waseda University, 2-2 Wakamatsu, Shinjuku, Tokyo, 162-8480 Japan; 2 Major in Integrative Bioscience and Biomedical Engineering, Graduate School of Advanced Science and Engineering, Waseda University, 2-2 Wakamatsu, Shinjuku, Tokyo, 162-8480 Japan; 3 Technology Center, Nihon Kohden Corporation, 1-31-4, Nishiochiai, Shinjuku, Tokyo, Japan

## Abstract

Automated blood cell counters can distinguish cells based on their size and the presence or absence of a nucleus. However, most vertebrates have nucleated blood cells that cannot be counted automatically. We established an alternative automatic method for counting peripheral blood cells by staining cells with the fluorescent dye acridine orange (AO) and analysing cell populations using flow cytometry (FCM). As promising new animal models, we chose *Xenopus laevis* and three inbred strains of *X. tropicalis*. We compared the haematological phenotypes, including blood cell types, cell sizes, cellular structure, and erythrocyte lifespans/turnover rate among *X. laevis* and the three inbred strains of *X. tropicalis*. Each cell type from *X. laevis* was sorted according to six parameters: forward- and side-scattered light emission, AO red and green fluorescence intensity, and cellular red and green fluorescence. Remarkably, the erythrocyte count was the highest in the *Golden* line, suggesting that genetic factors were associated with the blood cells. Furthermore, immature erythrocytes in anaemic *X. laevis* could be separated from normal blood cells based on red fluorescence intensity. These results show that FCM with AO staining allows for an accurate analysis of peripheral blood cells from various species.

## Introduction

The determination of haematological variables is essential for both laboratory and clinical research and is a routine technique for analysis in humans, fisheries, and domestic animals. Recently, haematological parameters have been recognized as a useful assessment tool for analysing sub-mammalian exotic animals, such as amphibians^1^. The study of the African clawed frog, *Xenopus laevis*, has led to many remarkable and vital contributions to cell and developmental biology. Its haematological parameters and morphology have been reported^2,3^, and in recent years, its complete genome was sequenced^4^. However, *X. laevis* has some disadvantages as an experimental model. For example, it is an allotetraploid species, and becomes sexually mature 10–24 months post metamorphosis^5^. In contrast, the western clawed frog *X. tropicalis* has a smaller diploid genome and a shorter generation time, making it advantageous to study the *X. tropicalis* over *X. laevis*. Moreover, the *X. tropicalis* genome has recently been fully sequenced^6^. However, the previously published haematological parameters of *X. tropicalis* cannot account for the differences between strains of *X. tropicalis*^7^.

The basic assays involved in haematological analysis, including blood cell counting, haemoglobin determination, the glass plate test for determining differential white blood cell count, and determination of the Wintrobe haematocrit or microhematocrit^8^, were established around 1900. Currently, the use of a haemocytometer for manually counting blood cells is a fundamental procedure for blood testing, and is required for counts in outlier samples containing leukocytes, platelets, and other cells. However, sampling a small number of cells to determine the concentration of a particular cell type is far too inaccurate to provide reproducible and reliable data for anything but major changes in the true count, making the haemocytometer insufficient for accurate blood cell counting^9^. To solve this problem, blood cell counts in mammals, including humans, are performed by an automatic cell counter that utilizes various features of blood cells, such as size and the presence or absence of a nucleus, for counting and sorting. However, because most sub-mammalian vertebrates have nucleated blood cells, an automated cell counter cannot be applied for such species. As a result, the counting of blood cells in these species currently relies upon the manual method that employs a haemocytometer under light microscopy. Although this method is easy to use with Shaw’s diluent solution, supravital stain^2^, or Natt and Herrick solution^10^, it remains difficult to distinguish between activated thrombocytes and lymphocytes. To develop an automated classification and counting system, we used *X. laevis* and *X. tropicalis* as animal models, since they possess nucleated blood cell types including erythrocytes, leukocytes, and thrombocytes, as well as their unclassified progenitors^2,11,12^. Additionally, using the T12 monoclonal antibody targeting *X. laevis* thrombocytes^13^, we were able to distinguish *X. laevis* thrombocytes from other types of blood cells.

Fluorescence analysis can be performed qualitatively using fluorescence microscopy or quantitatively using flow cytometry (FCM). Fluorochromes can bind to DNA and RNA separately, thereby enabling independent labelling. For example, murine hematopoietic stem cells (HSCs) were stained using Hoechst 33342, a DNA-bound fluorochrome that emits two wavelengths (Hoechst Blue and Hoechst Red), to distinguish side-population (SP) cells^14^. Cell classification by supravital cell staining with acridine orange (AO) was first reported in the 1960s^15,16^. Classically, this metachromatic fluorochrome has been used to rapidly stain DNA and RNA independently, based on AO emissions of green fluorescence upon binding to double-stranded DNA, and red fluorescence upon binding to single-stranded RNA. A previous study evaluated lymphocytes from human peripheral blood (PB) by fluorescent microscopy using AO-stained preparations^17^. Nowadays, it is known that AO accumulates within lysosomes and azurophilic granules of living cells and emits red fluorescence^18–20^. This type of staining enables clinicians to diagnose patients with chronic lymphocytic leukaemia, pertussis, hypogammaglobulinemia, acute leukaemia, uraemia, and other malignancies, as well as to distinguish human reticulocytes from erythrocytes. Moreover, morphological abnormalities present in human erythrocytes, such as red blood cell fragments and large platelets can be detected via FCM with AO staining^21^. In this study, six parameters were used to identify blood cell types: forward-scattered light (FSC), side-scattered light (SSC), nucleic acid and intracellular granule information obtained from green (F530) and red (F695) fluorescence intensity, cellular red fluorescence intensity (F695/FSC), and cellular green fluorescence intensity (F530/FSC). This analysis method has the potential to classify and separate blood cells, and to identify haematological abnormalities in sub-mammalian species where automated blood counting is as yet not possible. Our study will also enable researchers to characterize the haematological features of various animals, including genetically modified frogs and fish.

## Results

### The molar extinction coefficient of haemoglobin in *X. laevis*

**Figure 1:**
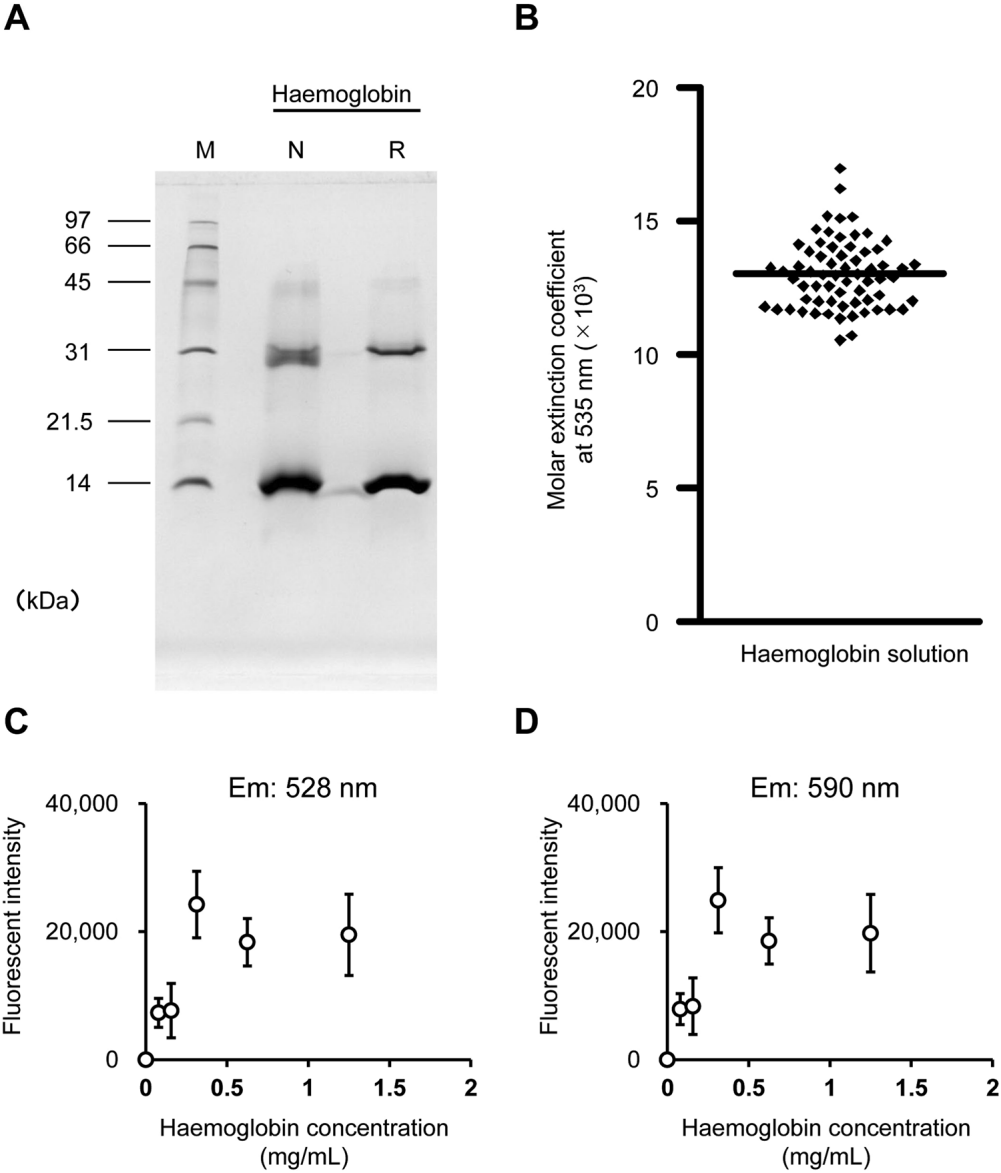
The molar extinction coefficient of sodium lauryl sulphate-haemoglobin (SLS-Hb). (**A**) *X. laevis* haemoglobin was purified, and SDS-PAGE was performed. Monomeric (14 kDa) and dimeric (30 kDa) Hbs were detected. M: Molecular marker; N: Non-reduction condition; R: Reduction condition. (**B**) The absorbance at 535 nm of SLS-Hb was measured and the molar extinction coefficient was calculated. Bar indicates the mean of the extinction coefficient (n = 66). (**C,D**) The fluorescence of purified haemoglobin was measured. (**C**) Excitation 485/20 nm, emission 528/20 nm. (**D**) Excitation 485/20 nm, emission 590/35 nm.

Purified haemoglobin fraction was subjected to SDS-PAGE and proteins of approximately 14 kDa and 30 kDa were detected (Fig. 1A). The haemoglobin complex was denatured and monomeric and dimeric haemoglobins could be visualised. After the purified haemoglobin was diluted and SLS solution was added, Abs_535_ was measured. The molar extinction coefficient of SLS-Hb was determined to be 13,010 L/mol/cm (Fig. 1B). There was no linear increase in the fluorescence intensity of haemoglobin proportional to its concentration (Fig. 1C,D).

### Optimal time course and AO concentration for staining blood cells

The relative fluorescence intensity (RFI; see Methods) rapidly increased during the first 10 min, then gradually increased until 120 min. There was no significant difference in the RFI between 15 and 120 min (Fig. S2A). When cells were incubated with different AO concentrations, the RFI increased in a concentration-dependent manner. However, at concentrations higher than 10 µg/mL, the number of cells tended to decrease, and at 20 µg/mL, the number of cells decreased significantly compared with the control group (without AO) (*p = *0.004) (Fig. S2B).

### Three populations were determined using green and red fluorescence intensity

**Figure 2:**
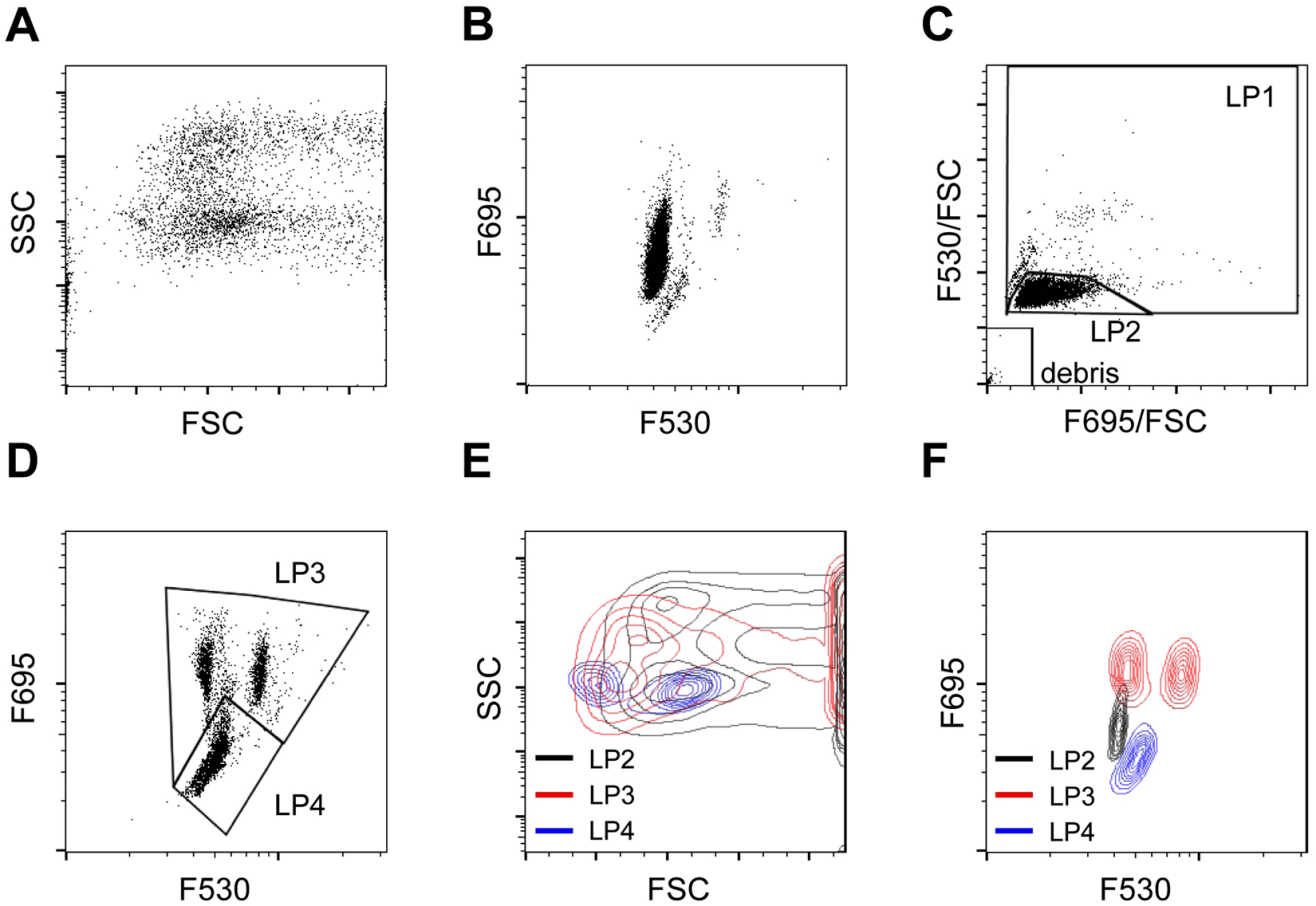
Scattergram analysis of peripheral blood from *X. laevis*. (**A**) Scattergram indicates forward-scattered light (FSC) and side-scattered light (SSC). (**B**) Scattergram indicates the F530 and F695 fluorescence intensity. (**C**) *X. laevis* blood cell population LP1, LP2, and debris fractions developed based on the cellular red fluorescence intensity (F695/FSC) and cellular red fluorescence intensity (F530/FSC). (**D**) LP3 and LP4 were determined based on the F695 fluorescence intensity. (**E**) LP2, LP3, and LP4 were identified based on FSC and SSC. LP2 and LP3 partially overlapped, and LP4 completely overlapped with LP2 and LP3. (**F**) LP2, LP3 and LP4 were determined based on the F530 and F695 fluorescence intensity. LP2: Erythrocytes (grey); LP3: Granulocytes (Red); LP4: Lymphocytes and Thrombocytes (Blue).

**Figure 3:**
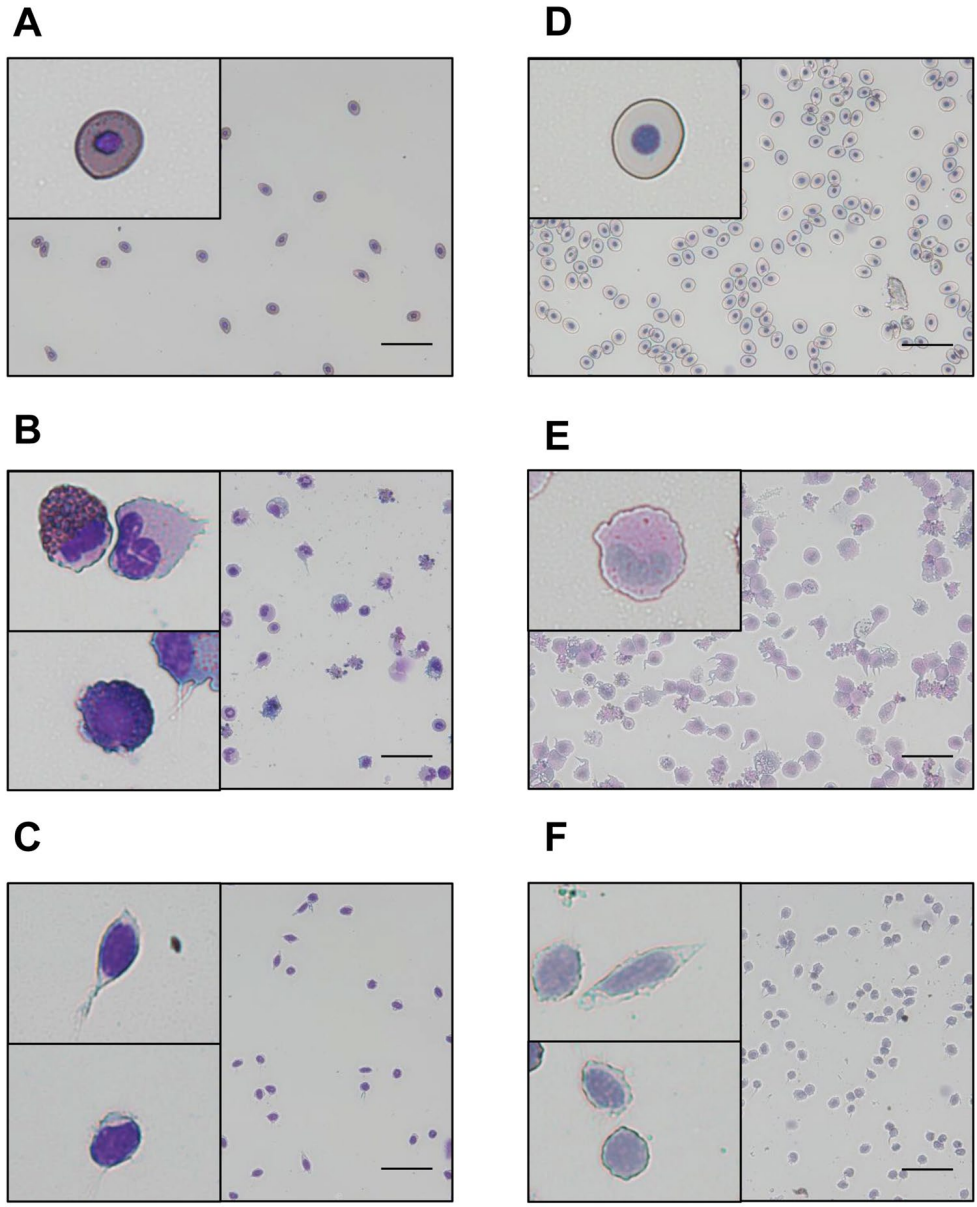
Morphology of LP2, LP3, and LP4 cells. Erythrocytes and leukocytes/thrombocytes separated from whole blood cells using the Percoll method were analysed and sorted. May-Grunwald Giemsa (MGG) and periodic acid-Schiff (PAS) staining were performed on the LP2, LP3, and LP4 fractions. (**A–C**) MGG staining. (**A**) LP2, (**B**) LP3, and (**C**) LP4. LP2 cells were mononuclear with a large cytoplasm. LP2 cells had eosinophilic cytoplasm. LP3 had segmented nuclei and a large cytoplasm. Cells with a basophilic cytoplasm were found in the LP3 fraction. LP4 cells were mononuclear with a small cytoplasm. (**D–F**) PAS staining. (**D**) LP2, (**E**) LP3, and (**F**) LP4. The LP2 and LP4 cells were negative for PAS staining, and LP3 cells were positive for PAS staining. Scale bars: 20 µm. LP2: erythrocyte; LP3: granulocyte; LP4: lymphocyte and thrombocyte (inset).

Each type of blood cell contained different amounts of green and red fluorescence intensity, based on FSC and SSC (Fig. 2A,B). Based on F695/FSC and F530/FSC, blood cells of *X. laevis* were subdivided into three fractions: LP1, LP2, and debris (Fig. 2C). Approximately 90% of the blood cells were present in the LP2 fraction. Based on the green and red fluorescence intensity, the LP1 fraction was further split into the LP3 and LP4 fractions (Fig. 2D). A scattergram was drawn for LP2, LP3, and LP4 based on FSC and SSC. LP2 showed a wide distribution and overlapped with the LP3 and LP4 fractions (Fig. 2E). On the basis of green and red fluorescence intensity, only the LP2 and LP3 partially overlapped (Fig. 2D). To characterize the cells in each fraction, FCM was performed after the separation of erythrocytes and leukocytes/thrombocytes. Erythrocytes were found in LP2, while leukocytes/thrombocytes were identified in the LP3 and LP4 fractions. We further analysed each fraction using MGG and PAS staining and obtained the following results. In the LP2 fraction, cells with an elliptical shape and large eosinophilic cytoplasm were found (Fig. 3A), indicating negative PAS staining (Fig. 3D). In the LP3 fraction, lobulated nuclei were eccentrically positioned and eosinophilic or basophilic granules were contained in some cells (Fig. 3B), indicating positive PAS staining (Fig. 3E). In the LP4 fraction, cells with a small size and poor cytoplasm were identified (Fig. 3C), indicating negative PAS staining (Fig. 3F). Although the number of erythrocytes and thrombocytes showed good correlation when staining with varying cell density, the number of leukocytes tended to decrease when cell density was increased (Fig. S2A–C). When blood cells were stained at a ratio of 1: 500, there was no cell population with high FSC (Fig. S3D); however when blood cells were stained at a ratio of 1:25, the cell population with high FSC appeared markedly (Fig. S3E).

### Thrombocyte separation

**Figure 4:**
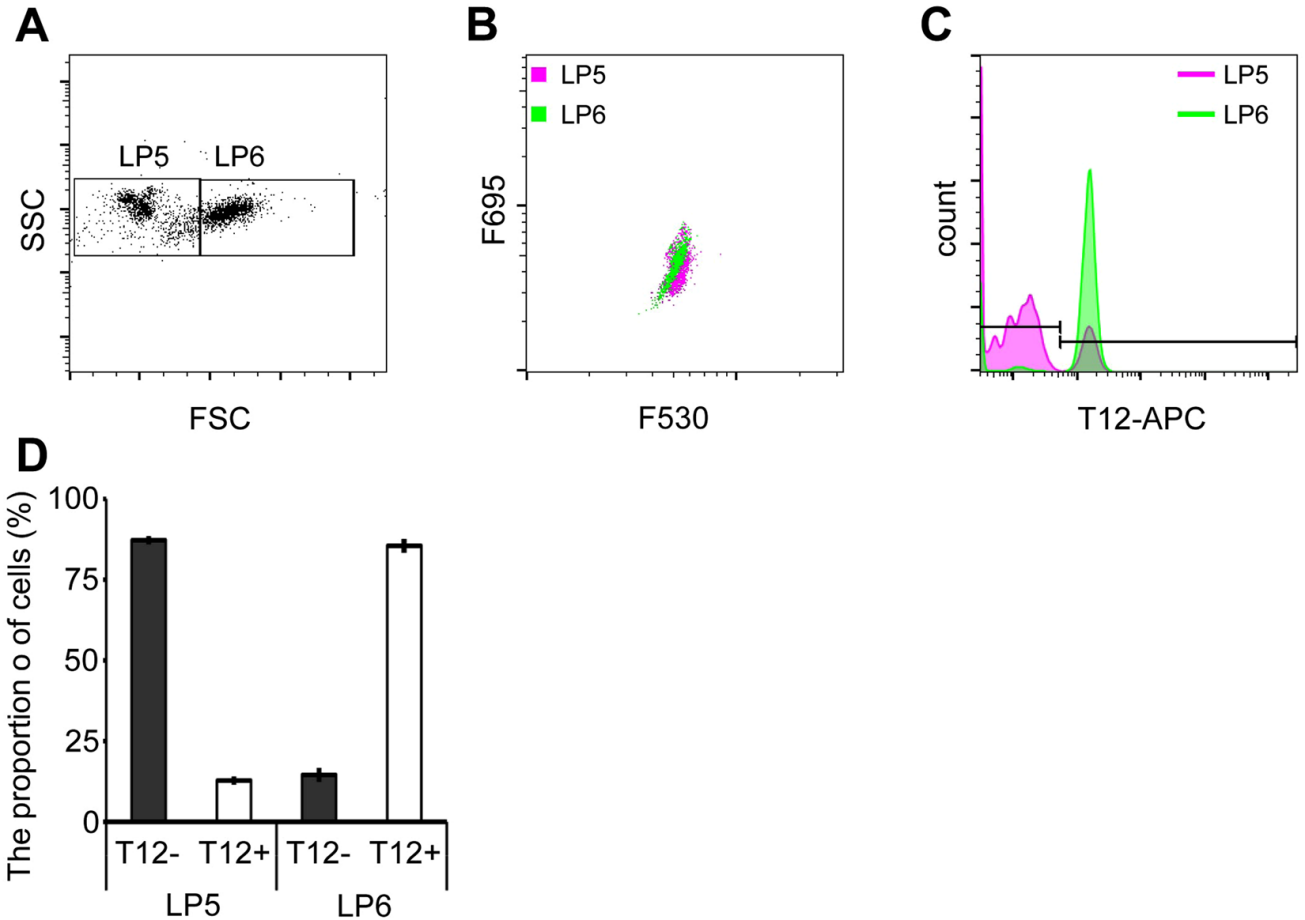
Separation of thrombocytes from the LP4 fraction. (**A**) The LP4 fraction was analysed using forward-scattered light (FSC) and side-scattered light (SSC), and categorized as LP5 and LP6. The FSC of LP6 was higher than that of LP5. (**B**) LP5 and LP6 were analysed using F530 and F695 fluorescence intensity. LP5 and LP6 overlapped. (**C,D**) LP5 and LP6 cells were immunostained with the T12 antibody and analysed by flow cytometry (FCM). T12-positive cells were mostly found in the LP6 fraction, although the LP5 fraction had T12-positive cells (approx. 10%).

To determine the proportion of thrombocytes, the cells in the LP4 fraction were further divided into two fractions based on their FSC and SSC parameters: LP5 and LP6 (Fig. 4A). The LP5 and LP6 fractions were then analysed based on the green and red fluorescence intensity in each fraction (Fig. 4B). Both LP5 and LP6 were subjected to immunostaining with T12, wherein approximately 90% of the LP6 cells tested positive for T12 (Fig. 4C,D).

### Analysis of *X. tropicalis* PB

**Figure 5:**
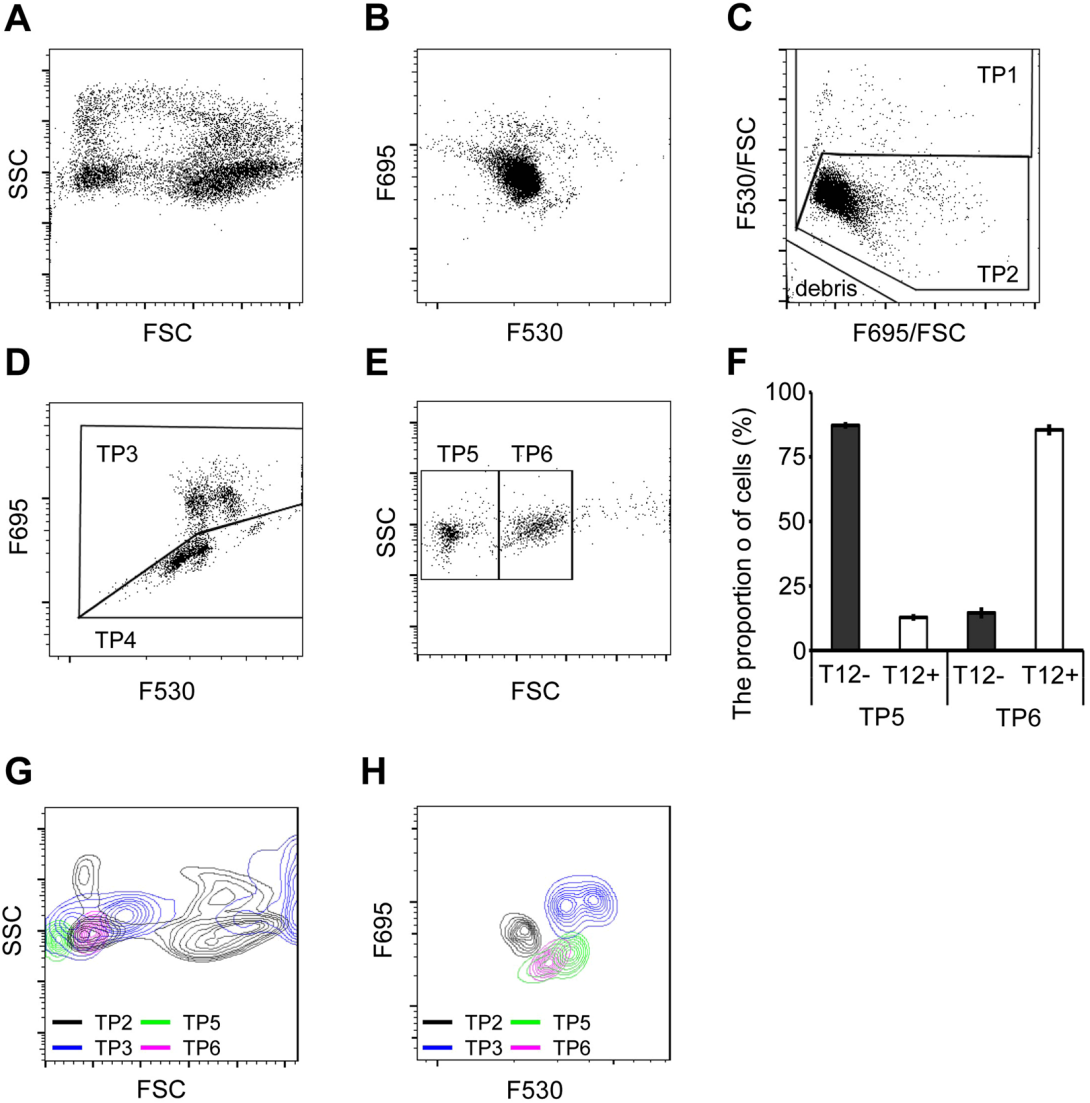
Scattergram analysis of peripheral blood (PB) from *X. tropicalis*. (**A**) Scattergram indicates FSC and SSC. (**B**) Scattergram indicates the F530 and F695 fluorescence intensity. (**C**) Blood cells were analysed for their cellular F695/FSC and F530/FSC intensities. The F530/FSC high and low populations were categorised as TP1 and TP2, respectively. (**D**) Based on F695 fluorescence intensity the TP1 fraction was separated into the TP3 (high intensity) and TP4 (low intensity) fractions. (**E**) Using FSC and SSC, the TP4 fraction was separated into TP5 (low FSC) and TP6 (high FSC). (**F**) TP5 and TP6 cells were immunostained with the T12 antibody and analysed by FCM. T12-positive cells were mostly found in the TP5 fraction, although the TP6 fraction had some T12-positive cells (approximately 10%). (**G**) TP2, TP3, TP5, and TP6 were developed using FSC and SSC. (**H**) TP2, TP3, TP5, and TP6 were analysed based on the F530 and F695 fluorescence intensity. TP2: Erythrocytes (grey); TP3: Granulocytes (Red); TP5: Lymphocytes (green); TP6: Thrombocytes (purple).

To investigate whether AO staining is applicable for diploid species, we analysed the PB of *X. tropicalis*. Each type of blood cell contained different green and red fluorescence intensity, based on FSC and SSC (Fig. 5A,B). Similar to that done for *X*. *laevis* blood cells, the *X*. *tropicalis* blood cells were fractionated into the TP1, TP2, and debris fractions (Fig. 5C), with TP1 being further sub-divided into the TP3 and TP4 fractions (Fig. 5D). Based on FSC and SSC, the TP4 fraction contained two additional fractions, TP5 and TP6 (Fig. 5E). Approximately 80% of TP6 cells were positive for T12 (Fig. 5F). Flow cytograms were drawn for the four fractions (TP2, TP3, TP5, and TP6) based on FSC, SSC, and the green and red fluorescence intensity. TP2 was found to be widely distributed and overlapped with TP3, TP5, and TP6 (Fig. 5G,H).

### Comparison between *X. laevis* and *X. tropicalis* PB

**Figure 6:**
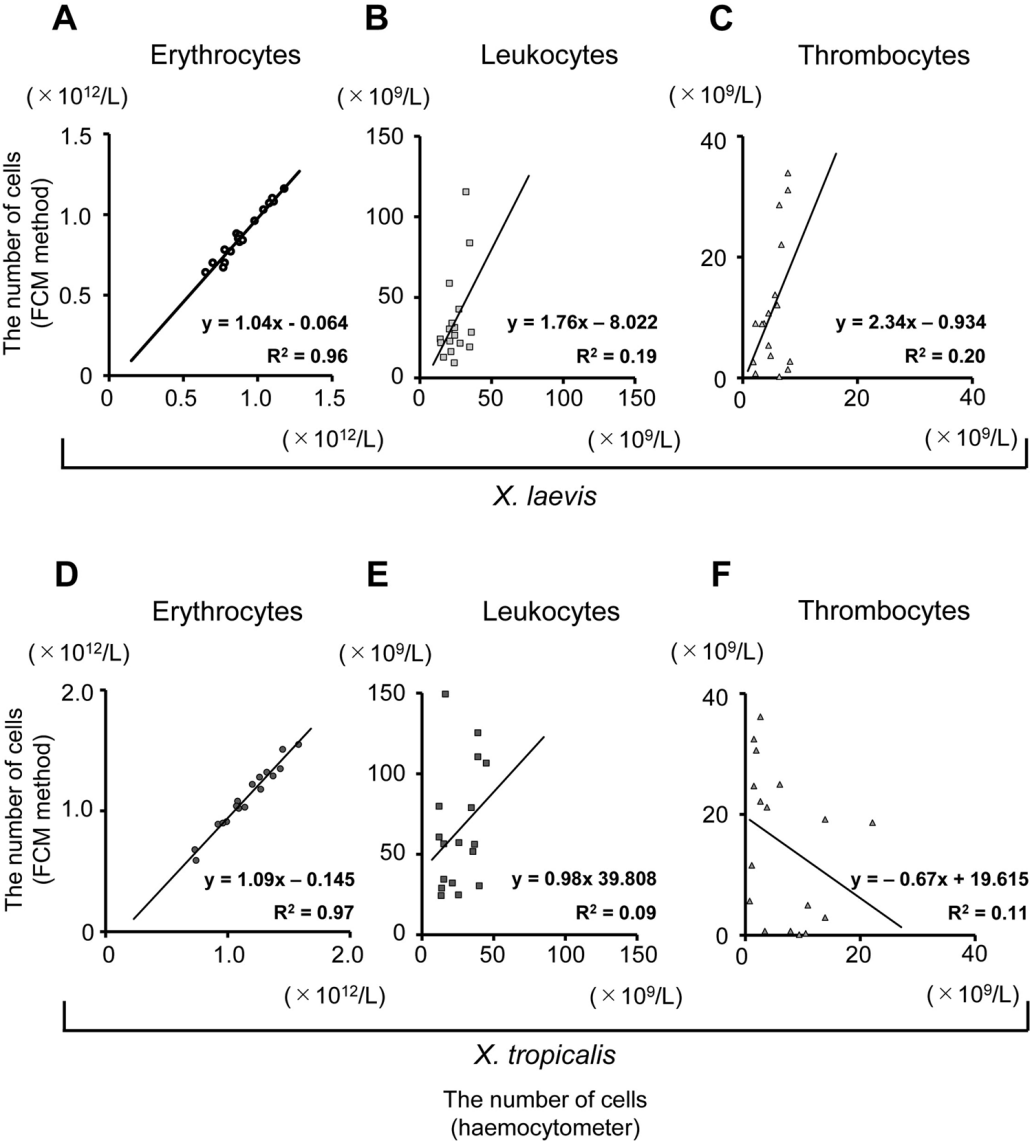
Comparison of haematological parameters between acridine orange (AO) staining and haemocytometer results. (**A–C**) Correlation between AO-staining and haemocytometer results in *X. laevis*. The number of (**A**) erythrocytes, (**B**) leucocytes, and (**C**) thrombocytes was identified. The number of leukocytes was determined using FCM, according to the sum of the number of granulocytes and lymphocytes. (**D–F**) Correlation between AO-staining and haemocytometer results in *X. tropicalis*. The number of (**D**) erythrocytes, (**E**) leucocytes, and (**F**) thrombocytes was determined.

We used seventeen samples each of *X. laevis* PB and *X. tropicalis* PB (from *Golden* [n = 5], *Nigerian H* [n = 6], and *Ivory Coast* [n = 6]) to determine the number of erythrocytes, leukocytes, and thrombocytes using the AO staining method. The results were compared with that obtained through counting with a haemocytometer (Fig. 6). The number of erythrocytes in *Golden* strain was found to be significantly higher than that in *X. laevis* (*p* = 0.001) and *Nigerian H* (*p* = 0.008). Additionally, the number of erythrocytes in *Ivory Coast* was significantly higher than that in *Nigerian H* (*p* = 0.026). The haemoglobin concentration in *Golden* was significantly higher than that in *X. laevis* (*p* = 0.001) and *Nigerian H* (*p* = 0.001) and haematocrit in *Golden* was significantly higher than that in *X. laevis* (*p* = 0.018), and *Nigerian H* (*p* = 0.002) (Supplementary Table 1). Additionally, the estimated erythrocyte lifespan in *X. tropicalis* was 280 days (Table 1 and Fig. S3), which is longer than that in *X. laevis*^22^. The rate of production of erythrocytes in *X. tropicalis* was calculated to be approximately 0.35% per day under normal conditions. Each type of blood cell was largely similar between *X. laevis* and *X. tropicalis*. However, the erythrocytes in *X. tropicalis* were smaller than those in *X. laevis* (Fig. S1).

**Table 1:** Blood volume and erythrocyte turnover in *X. laevis* and *X. tropicalis*.

	*X. laevis*	*X. tropicalis*
	Wild type	Golden
Blood volume (µL)	4,000	770
Erythrocyte lifespan (days)	220	280
Turnover rate (%)	0.45	0.35

### Analysis of anaemia model and immature erythrocytes

**Figure 7:**
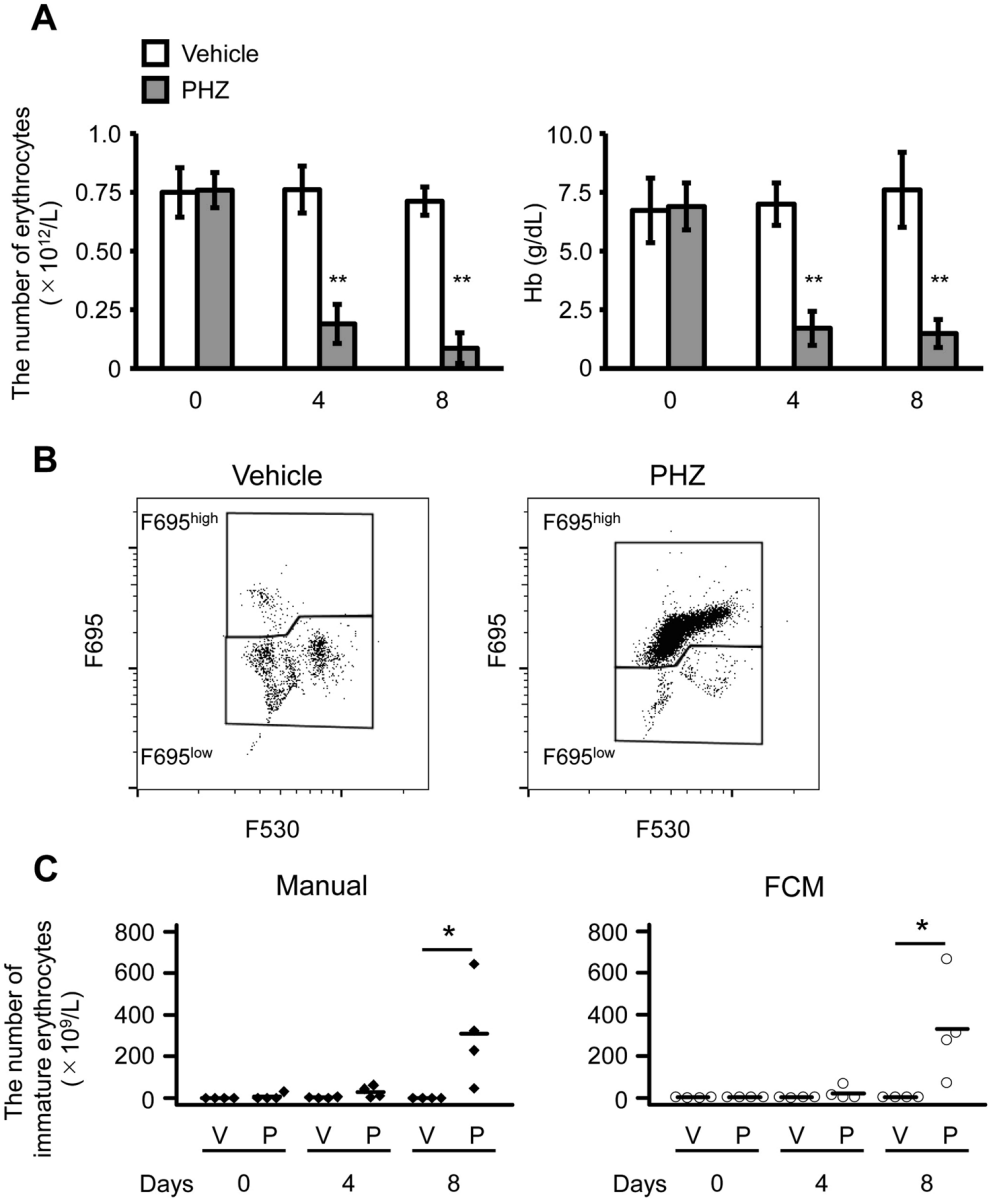
Analysis of peripheral blood derived from anaemic *X. laevis*. (**A**) Erythrocyte counts (left panel) and haemoglobin (Hb) concentration (right panel) in peripheral blood after the administration of phenylhydrazine (PHZ; 25 mg/kg body weight, intraperitoneally, day 0). All values are shown as the mean ± SD. **p < 0.01 vs control (n = 4). (**B**) The LP3 fraction was analysed to determine the F530 and F695 fluorescence intensity. The F695^high^ population of the LP3 fraction was markedly increased after PHZ administration. (**C**) Comparison of immature erythrocyte counts between the manual method (left panel) and flow cytometry (right panel). *p < 0.05 vs. control (n = 4).

Four days after PHZ administration, the peripheral erythrocyte count and the Hb concentration significantly decreased to 25% (*p* = 0.0003) and 24% (*p* = 0.0002), respectively, compared with the control group at day 4. Furthermore, on day 8, the peripheral erythrocyte counts dramatically decreased (*p* = 0.0001) compared with the control group at day 8. However, 8 days after PHZ administration, although the concentration of Hb was dramatically lower (*p* = 0.001) than that in the control group, it recovered slightly, but not significantly, compared with day 4 (Fig. 7A). Peripheral blood was stained with AO and subjected to FCM; cells showing intermediate FSC cells and high red fluorescence intensity were increased in anaemic PB (Fig. S4B). In the LP3 fraction, the F695^high^ fraction was markedly increased (Fig. 7B), and the cells did not display the characteristics of mature erythrocytes (LP2) or granulocytes (LP3) (Fig. S2C). The F695^high^ fraction was sorted and stained with *o-*dianisidine; cells that were *o-*dianisidine positive had a high nucleic/cytoplasm (N/C) ratio (Fig. S2D). Immature erythrocyte counts through manual counting and FCM were consistent, with the number of erythrocytes in the PHZ treated group being significantly higher than that in the control group on day 8 (*p* = 0.047, manual; *p* = 0.037, FCM).

## Discussion

We attempted to separate the blood cell types of *X. laevis* based on their FSC and SSC profiles and green and red fluorescence intensity. The RFI increased in a concentration-dependent manner, whereas in a high concentration of AO solution, the number of cells decreased. Since 5 µg/mL of AO solution was excellent in staining and did not decrease the number of cells, we considered that to be the optimal concentration when staining *Xenopus* blood cells. Furthermore, the reaction time was inferred to be stable for at least 10 minutes to 60 minutes. Therefore, the optimal condition was considered as 1 µL of PB diluted 500 times with 5 µg/mL AO with staining up to 10–60 minutes. In addition, since human erythrocytes show no detectable fluorescence^23^, it is thought that interference by haemoglobin does not occur. The cells in LP1 had abundant haemoglobin in the cytoplasm, and the cells in LP2 had an eosinophilic cytoplasm, determined by MGG staining. Furthermore, the LP2 cells were mononuclear, had a large cytoplasm, and were negative for PAS staining. These results indicated that LP2 was the erythrocyte fraction. The cells in the LP3 fraction showed lobulated nuclei that were eccentrically positioned, and positive PAS staining. Neutrophils in *X. laevis* show positive PAS staining^2^; thus, LP3 was identified as the granulocyte fraction. T12 specifically reacts to spindle-shaped thrombocytes^13^; hence the LP6 was identified as the thrombocyte fraction. Additionally, the cells in LP5 were smaller than those in LP6, mononuclear, and stained negative for PAS. The presence of T12 negative cells in the LP5 fraction suggested that two types of lymphocytes existed in the PB^2^. Thrombocytes were 6.7–15.6 µm long, and the large lymphocytes had a diameter of 10.0–11.0 µm. T12-positive cells were identified in the LP6 fraction. Since thrombocytes are elliptical in shape, it is likely that two sizes of thrombocytes were present in the PB. Erythrocytes are also elliptical in shape, and the FSC of erythrocytes showed a wide distribution. As the lymphocytes were small, we assumed that LP5 was the lymphocyte fraction.

The morphology of blood cells in *X. tropicalis* was similar to that in *X. laevis*. TP2 was identified as the erythrocyte fraction because it accounted for approximately 90% of the blood cells. Since TP5 contained T12-negative cells with low FSC (approximately 90%), while the cells in TP6 were T12-positive (approximately 80%) and had an intermediate FSC. We, therefore, assumed that TP5 and TP6 were the thrombocyte and lymphocyte fractions, respectively. TP4 had a high red fluorescence intensity; we, therefore, propose that it contained the granulocyte fraction. As the proportion of blood cells to the AO staining solution increases, the staining property of the intracellular granule is inferior and the red fluorescence is attenuated. This could be the reason why the white blood cell count is estimated to be low. When 1 µL of PB is diluted 25 times with 5 µg/mL AO solution, high FSC cells were contaminated in the F695^low^ fraction (LP3). The result suggests that the cellular granules and/or lysosomes are insufficiently stained and cannot be distinguished from thrombocytes and lymphocytes.

We observed major differences in the number of erythrocytes, the haemoglobin concentration, and haematocrit between the three inbred strains (*Golden*, *Nigerian H*, and *Ivory Coast*) of *X. tropicalis*. The results indicated that there was a difference in oxygen supply to the peripheral tissues between the *X. tropicalis* strains. Russell *et al*. have reported that complete blood counts differed between strains in mice, and also found chance fixation in different genotypes^24^. The *Golden* line was characterized by rapid growth, and the *Nigerian H* strain differed from the *Ivory Coast* strain^5^, suggesting that genetic factors might be associated with the differences in the number of blood cells in *X. tropicalis* strains.

In anaemic rats induced by PHZ, erythropoiesis is enhanced, and reticulocytes—which are immature erythrocytes —increase in the PB^25^. In our study, in anaemic *X. laevis* induced by PHZ, immature erythrocytes with haemoglobin and high N/C ratio were significantly increased in PB. Furthermore, after staining PB with AO, immature erythrocytes could be distinguished from other normal blood cells. Thus, FCM-based cell counting was confirmed to be consistent with the manual method and was effective without having to use any specific cell surface markers. Additionally, the erythrocyte cell size in *X. laevis* was larger than that observed in *X. tropicalis*, and the FSC values associated with *X. laevis* blood cells were higher than those associated with *X. tropicalis*. These results suggest a high correlation between blood cell size and the FSC value, despite the differences in the origin and the nuclear ploidy of the blood cells. Generally, immature cells exhibit high N/C ratios and a high basophilic cytoplasm, owing to the abundance of mRNA. Moreover, HSCs exhibit a small cell size and basophilic cytoplasm, indicating that measurement of cell size and RNA content by AO staining and FCM using our method might be useful for the analysis of hematopoietic stem/progenitor cells across species. On the other hand, membrane permeation treatment under low pH is required for the differential staining of DNA and RNA by using AO^26^. Furthermore, higher order RNA structures may be stained as double-stranded nucleic acids, due to which double-stranded DNA and RNA may not be separated. Future studies are required to devise a method for determining the exact amount of DNA and RNA without false-positive results caused by the state of nucleic acids. In conclusion, our findings show that the use of AO staining to distinguish different blood cells by FCM was more effective than standard methods based on its ability to separate nucleated erythrocytes and thrombocytes. This was confirmed by the high correlation with results obtained using a haemocytometer. Furthermore, our results revealed that the AO method enabled accurate cell size fractionation according to FSC, indicating that this method might also be useful for the study of stem cell differentiation and the identification of blood diseases such as leukaemia. In this article, we successfully exploited this protocol as a phenotyping method in a genetically modified frog model.

## Materials and Methods

### Animal models and PB preparation

Wild-type male African clawed frogs (*X. laevis*; weighing 20–30 g) were purchased from Ohuchi Aquatic Animal Supply (Misato, Saitama, Japan). Frogs were housed in a plastic case under a 12-h/12-h light/dark cycle at room temperature (22 °C) with constant running water, before initiation of experiments. The *Golden* line, *Nigerian H* line, and *Ivory Coast* line of *X. tropicalis* were provided by the Amphibian Research Center (Hiroshima University) through the National Bio-Resource Project (https://home.hiroshima-u.ac.jp/amphibia/xenobiores_en/iweb_en/Top.html) of the Ministry of Education, Culture, Sports, Science, and Technology (MEXT), Japan. *X. tropicalis* were maintained in dechlorinated tap water at 25 °C. Blood samples from *X. laevis* were obtained by cardiac puncture with a 27-gauge needle attached to a capillary tube (Drummond Scientific, Broomall, PA, USA) or a 1-mL syringe (Terumo, Tokyo, Japan) coated with anti-coagulant EDTA-2Na. *X. tropicalis* PB samples were obtained by cardiac puncture with 29-gauge insulin syringes (SS-10M2913A; Terumo) coated with EDTA-2Na (Supplementary Video 1). Blood was treated with 0.8× Dulbecco’s modified phosphate-buffered saline (dDPBS) to remove Mg^2+^ and Ca^2+^ ions to prevent coagulation during dilution of the whole blood, as necessary. A standard saline solution was used to dilute samples to adjust for amphibian osmotic balance.

### *Xenopus* haemoglobin purification

*Xenopus* haemoglobin (Hb) was extracted by sonication, as previously described^27^. Briefly, blood was centrifuged at 300 × g for 30 min at room temperature (RT). The erythrocyte pellet was washed three times in equal volume of dDPBS and centrifuged at 1,000 × g for 30 min. Then, the erythrocyte pellet was suspended in double the volume of 10 mM Tris-HCl (pH 8.0) and sonicated on ice. Erythrocyte homogenates were incubated at 60 °C for 1 h, and then centrifuged at 2,000 × g for 1 h. An equal volume of 10 mM Tris-HCl (pH 8.0) was added to the supernatant and centrifuged at 2,000 × g for 1 h. The supernatant was dialyzed against 10 mM Tris-HCl (pH 8.0) and then centrifuged at 5,000 × g for 20 min. Anion exchange chromatography (Q Sepharose High Performance; GE Healthcare, Milwaukee, WI, USA) was performed on the Hb solution. Purified Hb was separated using an SDS-PAGE and revealed and stained with Coomassie Brilliant Blue.

### The molar extinction coefficient of *Xenopus* haemoglobin

Purified *Xenopus* Hb was quantified by a haematology analyser (Celltac α; Nihon Kohden, Tokyo, Japan) to determine tentative concentration. The Hb solution was diluted with sodium lauryl sulphate (SLS) solution, as previously described^28^, and its absorbance at 535 nm (Abs_535_) was measured. The molar extinction coefficient was calculated using the following equation (1):

1 $${{\rm{\varepsilon }}}_{535}=\frac{{{\rm{Abs}}}_{535}\times {\rm{M}}}{{\rm{c}}({\rm{g}}/{\rm{dL}})\times 10}.$$

where ε_535_ = molar absorptivity at 535 nm; M = relative molecular mass of haemoglobin, derived from 66386.4/4; and c = concentration of Hb solution; and 10 = conversion factor (g/L to g/dL).

### Concentration-fluorescence intensity curves of purified haemoglobin

Purified *Xenopus* Hb was diluted and dispensed into 96-well black polystyrene plate (Costar #3915; Corning, Inc., Corning, NY, USA). The fluorescence intensity was measured at an excitation wavelength of 485 nm and an emission wavelength of 528 nm or 590 nm by a microplate reader (Power Scan HT; Dainippon Sumitomo Pharma, Osaka, Japan).

### Blood cell analysis

Based on the previous report, the number of erythrocytes, leucocytes, and thrombocytes was counted with a haemocytometer after diluting blood at a ratio of 1:150 in Shaw’s diluting solution^2^. We differentiated erythrocytes, leucocytes, and thrombocytes from cell morphology. Haematocrit was measured after blood-sample centrifugation in a swing-out rotor at 2,500 × *g* for 5 min in capillary tubes. As described by Oshiro *et al*.^28^, the haemoglobin concentration was measured by gently mixing 1 µL blood with 250 µL working SLS solution and measuring the Abs_535_. The following equation (2)^29^ calculation was used to calculate haemoglobin concentration.

2 $${\bf{c}}({\bf{g}}/{\bf{d}}{\bf{L}})=\frac{{\bf{A}}{\bf{b}}{{\bf{s}}}_{535}\times {\bf{M}}\times {\bf{F}}}{{{\boldsymbol{\varepsilon }}}_{535}\times {\bf{l}}\times 10}.$$

where Abs_535_ = absorbance of the solution at λ = 535 nm; M = relative molecular mass of haemoglobin, derived from 66386.4/4; F = dilution factor; ε_535_ = molar absorptivity; l = light path (cm); and 10 = conversion factor (g/L to g/dL). For a dilution factor (F) of 251, light path of 1.0 cm, and ε_535_ of 13.01:

3 $${\rm{c}}({\rm{g}}/{\rm{dL}})=31.967\times {{\rm{Abs}}}_{535}$$

### Cytological analysis

Cytospin preparations of *X. laevis* and *X. tropicalis* blood cells were stained using May-Grunwald Giemsa solution (MGG; Wako, Osaka, Japan), as previously described^30^. *O*-dianisidine staining was performed as follows: samples were fixed for 5 min in methanol, incubated in 0.1% *o-*dianisidine and 3% hydrogen peroxide for 1.5 min, washed in running water, and counterstained with Giemsa solution^31^. For periodic acid-Schiff (PAS) staining, cells were fixed in 10% formalin-methanol solution for 30 min at room temperature, and washed with tap water. The slides were incubated in 1% sodium periodate for 5 min, and twice washed in distilled water. Glass slides were incubated in the Schiff reagent (Wako, Osaka, Japan) for 15 min at room temperature and then placed in Gill’s haematoxylin solution (Muto Chemical, Tokyo, Japan) for 10 min, washed with tap water, and examined using light microscopy (model BX51; Olympus, Tokyo, Japan).

### Concentration-intensity curves of blood cells with AO staining

We prepared 0.1–20 µg/mL AO in 10 mM HEPES buffer (pH 7.4) containing 117 mM NaCl (diluted HEPES buffered saline; dHBS). Blood cells (2 × 10^6^ cells) were incubated with 1 mL of each concentration of AO solution for 15 min in the dark at room temperature and washed with dHBS twice. Blood cells were suspended with 1 mL of dHBS. Each cell count was performed using a hemocytometer after diluting cells at a ratio of 1:10 in Shaw’s diluting solution. 100 µL of cell suspension was dispensed into each well of 96-well black polystyrene plate. The fluorescence intensity was measured at an excitation wavelength of 485 nm and an emission wavelength of 528 nm using a microplate reader. The relative fluorescence intensity (RFI) was calculated as the mean fluorescence intensity of the sample/mean fluorescence intensity of control cells.

### Time-intensity curves of blood cells with AO staining

PB (1 μL) was incubated with 500 µL of 5 µg/mL AO in HBS for 0–120 min in the dark at room temperature and washed with dHBS twice. Blood cells were suspended with 1 mL of dHBS. Each cell count was measured using a haemocytometer after diluting cells at a ratio of 1:100 in Shaw’s diluting solution. 200 µL of cell suspension was dispensed into each well of a 96-well black polystyrene plate. The fluorescence intensity was measured at an excitation wavelength of 485 nm and an emission wavelength of 528 nm by a microplate reader, and the RFI was calculated as above.

### FCM analysis

PB (1 μL) was incubated with 5 µg/mL AO in dHBS for 15 min in the dark at room temperature. We used blue light (488 nm) for fluorescence excitation and measure cell fluorescence in green (F530, 530/20 nm) and red (F695, 695/40 nm) light wavelength. Previously it was reported that the amount of DNA was determined by green fluorescence, and the amount of RNA by red fluorescence, and the cellular RNA concentration (CRc) and cellular DNA concentration (CDc) values could be evaluated by calculating the ratio between the RNA and FSC or DNA and FSC characteristics of each event, respectively^21^. Based on this report of, we defined the cellular red and green fluorescence intensity as red fluorescence intensity per FSC (F695/FSC) and green fluorescence intensity per FSC (F530/FSC), respectively. As the measurement was carried out using linear FSC with a linear scale (FSC-lin), Log (FSC-lin) was determined before F695/FSC and F530/FSC calculations. PB was diluted at a ratio of 1:25–1:2,500 in 5 µg/mL AO in dHBS and incubated for 15 min in the dark at room temperature. Blood cells were incubated with T12 antibodies^13^ for 30 min and washed with dDPBS containing 2% foetal calf serum (FCS) and 2 mM EDTA, followed by incubation with goat anti-mouse IgG-allophycocyanin conjugate, (Abcam, Cambridge, UK) for 30 min. Cells were analysed and sorted by fluorescence-activated cell sorting (FACS) using a FACS Aria II system (BD Biosciences, San Jose, CA, USA). All data were recorded using FACS Diva 6.1 software (BD Biosciences) and flow cytograms were generated using FlowJo v10.4.2. Mouse IgG1 (Bay Bioscience, Kobe, Japan) was used as isotype control for T12 antibodies.

### Complete blood cell count by FCM method

The absolute number of blood cells was calculated using the following equation (4):

4 $${\rm{X}}={\rm{a}}\times {\rm{b}}$$

where *a* is the total cell number (×10^12^/L) in PB measured using the haemocytometer, and *b* is the ratio of the target cell (erythrocyte: LP2 or TP2, granulocyte: LP3 or TP3, lymphocyte: LP5 or TP5, thrombocyte: LP6 or TP6) to the total cells excluding the debris fraction. The number of leucocytes is the sum of granulocyte and lymphocyte counts.

### Separation of leukocytes/thrombocytes from whole blood

Blood samples were layered onto a density gradient of 70% (v/v) Percoll (GE Healthcare, Milwaukee, WI, USA), buffered with dDPBS, and centrifuged at 500 × *g* for 15 min at room temperature. Leukocytes and thrombocytes were washed three times with dDPBS, and incubated in a solution containing T12 antibodies and AO. Stained leukocytes and thrombocytes were analysed and sorted using the FACS Aria II system (BD Bioscience).

### Lifespan of erythrocytes

Circulating erythrocytes were labelled *in vivo* with *N-*hydroxysulfosuccinimide biotin esters for labelling cell surface proteins (EZ-Link Sulfo-NHS-LC-biotin; Pierce Biotechnology, Waltham, MA, USA). Activated biotin solution (25 µL) in dDPBS was introduced by the intracardiac administration. Collected blood cells were washed with dDPBS containing 2% FCS and 2 mM EDTA, and incubated with Streptavidin Alexa-Fluor 488 Conjugate (Life Technologies, Carlsbad, CA, USA). The analysis of blood cells and the calculation of the half-life of erythrocytes were performed as previously described^22^.

### Induction of haemolytic anaemia by phenylhydrazine administration

Phenylhydrazine (PHZ; 25 mg/kg body weight; Sigma Aldrich, St Louis, MO, USA), in 100 µL dDPBS, was administered to male *X. laevis* frogs (n = 4) by intraperitoneal injection. As a control, 100 µL dDPBS was injected (n = 4)^32^. PB cells were collected by heart puncture after 0, 4, and 8 days. Blood cell count, haematocrit, haemoglobin concentration measurement, cytological analysis, and FCM analysis were performed as described above. Immature erythrocyte counts were calculated using the following equation (5):

5 $${\rm{Y}}={\rm{a}}\times {\rm{b}}\times {{\rm{10}}}^{{\rm{3}}}$$

where *a* is the total cell number (×10^12^/L) in PB, and *b* is the ratio of mature erythrocytes in the sample as determined by cytological analysis (manual) or FCM.

### Statistical analysis

Results are presented as the mean ± standard deviation. Data were analysed by one-way analysis of variance (ANOVA), and differences between the means were analysed using the Tukey-Kramer multiple-comparison test or Student’s *t-*test. Statistical analyses were performed using SPSS v. 24. A beeswarm plot was produced using the R package beeswarm.

### Study approval

All methods were performed in accordance with the relevant guidelines and regulations. All animal studies were approved by the Regulations for Animal Experimentation at Waseda University, Tokyo, Japan (2016–A001, 2016–A002, 2016–A003, 2017–A001, 2017–A002, 2017–A003, 2018-A076, 2018-A077, and 2018-A078).

## Electronic supplementary material

Supplementary information

Supplementary Video 1
