# Supplementary material for: Flow cytometric analysis of Xenopus laevis and X. tropicalis blood cells using acridine orange
Source: Sci Rep. 2018 Nov 2;8:16245. doi: 10.1038/s41598-018-34631-0 (PMC6214894; doi:10.1038/s41598-018-34631-0)
Supplement: Supplementary file 1 — Supplementary information [file 41598_2018_34631_MOESM1_ESM.pdf]

Supplementary Information

Flow cytometric analysis of *Xenopus laevis* and *X. tropicalis* blood cells using acridine orange

Kei SATO[1, 2], Azusa UEHARA[2], Sayaka KINOSHITA[2], Ikki NOMURA[2], Minami YAGI[2], Yuta TANIZAKI[2], Yu MATSUDA-SHOJI[2], Atsushi MATSUBAYASHI[2], Nobuyasu ENDO[1], Yutaka NAGAI[3], Takashi KATO[1, 2]

[1] Faculty of Education and Integrated Arts and Sciences, Waseda University, 2-2 Wakamatsu, Shinjuku, Tokyo 162-8480, Japan

[2] Major in Integrative Bioscience and Biomedical Engineering, Graduate School of Advanced Science and Engineering, Waseda University, 2-2 Wakamatsu, Shinjuku, Tokyo, 162-8480, Japan

[3] Technology Center, Nihon Kohden Corporation, 1-31-4, Nishiochiai, Shinjuku, Tokyo, Japan.

*Author for correspondence: Takashi Kato

Integrative Bioscience and Biomedical Engineering, Graduate School of Advanced Science and Engineering, Waseda University

2-2 Wakamatsu, Shinjuku, Tokyo 162-8480, Japan

Phone: +81 (3) 5369-7309, Fax: +81 (3) 3355-0316, Email: tkato@waseda.jp

Supplementary Figure S1

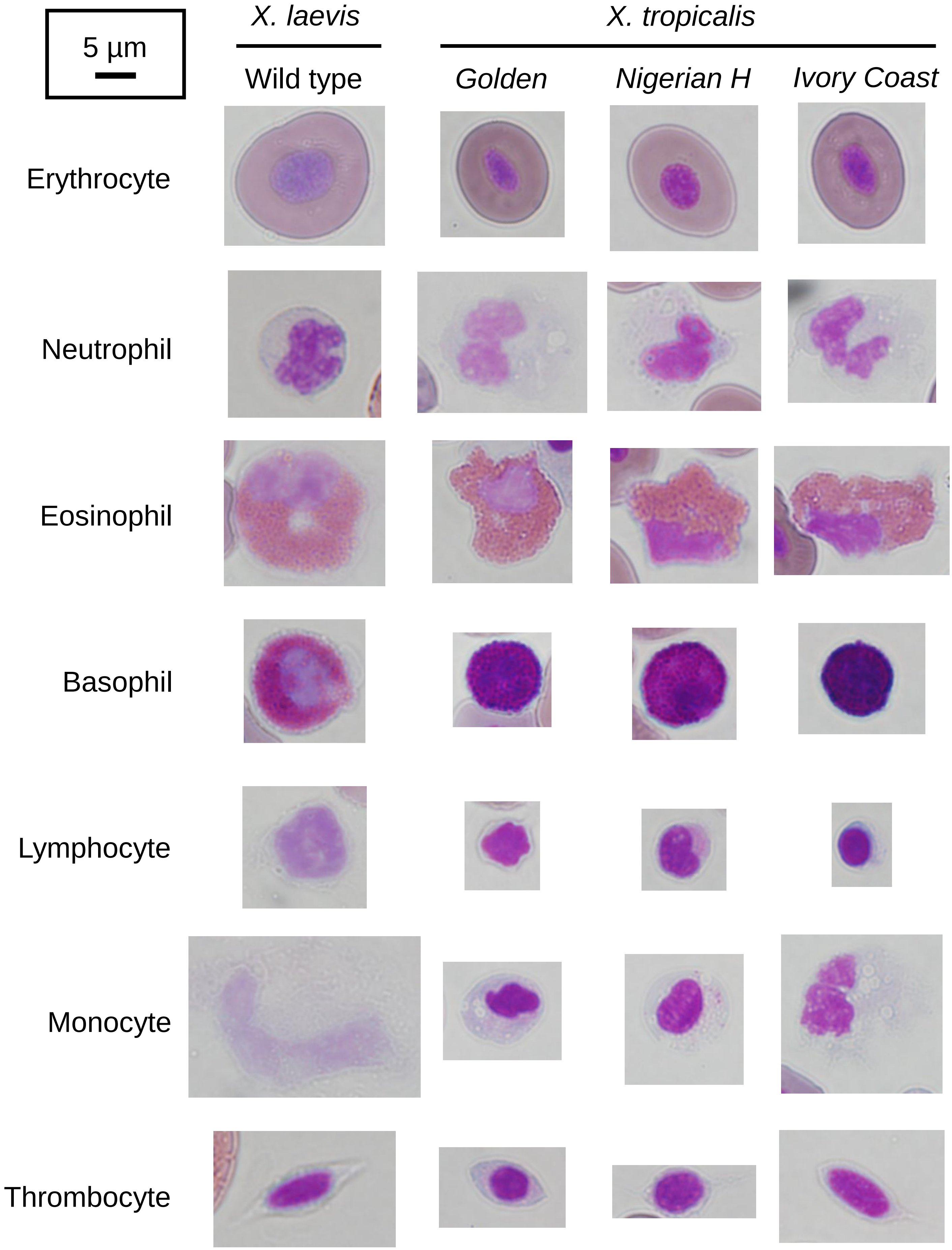

Supplementary Figure S1. Blood cells morphology of *Xenopus laevis* and *X. tropicalis*.
May-Grunwald Giemsa (MGG) stained peripheral blood cytospin. Each blood cell type was distinguished based on morphology.

Supplementary Figure S2

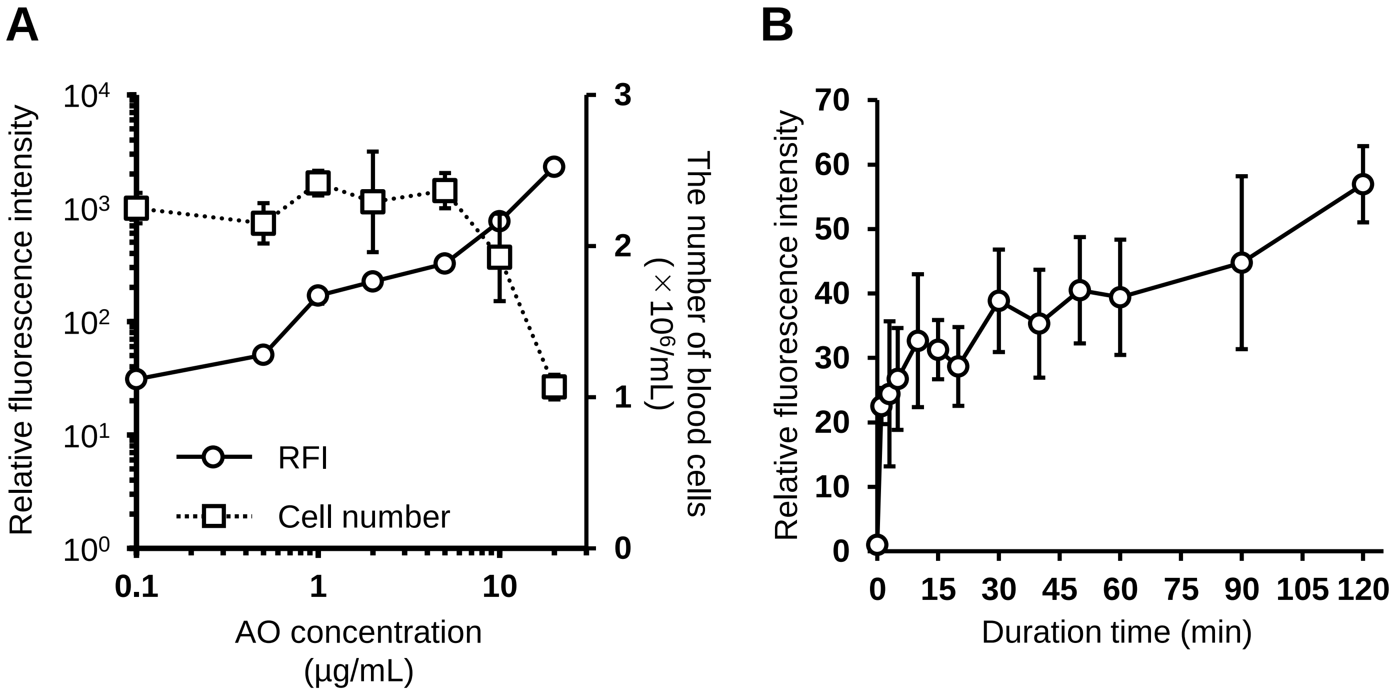

Supplementary Figure S2. The stability of acridine orange staining.
A: Concentration-intensity curves of blood cells. The solid line with circular symbols indicates the relative fluorescent intensity (RFI), and the dashed line with square symbols indicates the number of the blood cells. B: Time-intensity curves of blood cells. The solid line indicates the RFI. All values are shown as the mean ± SD.

Supplementary Figure S3

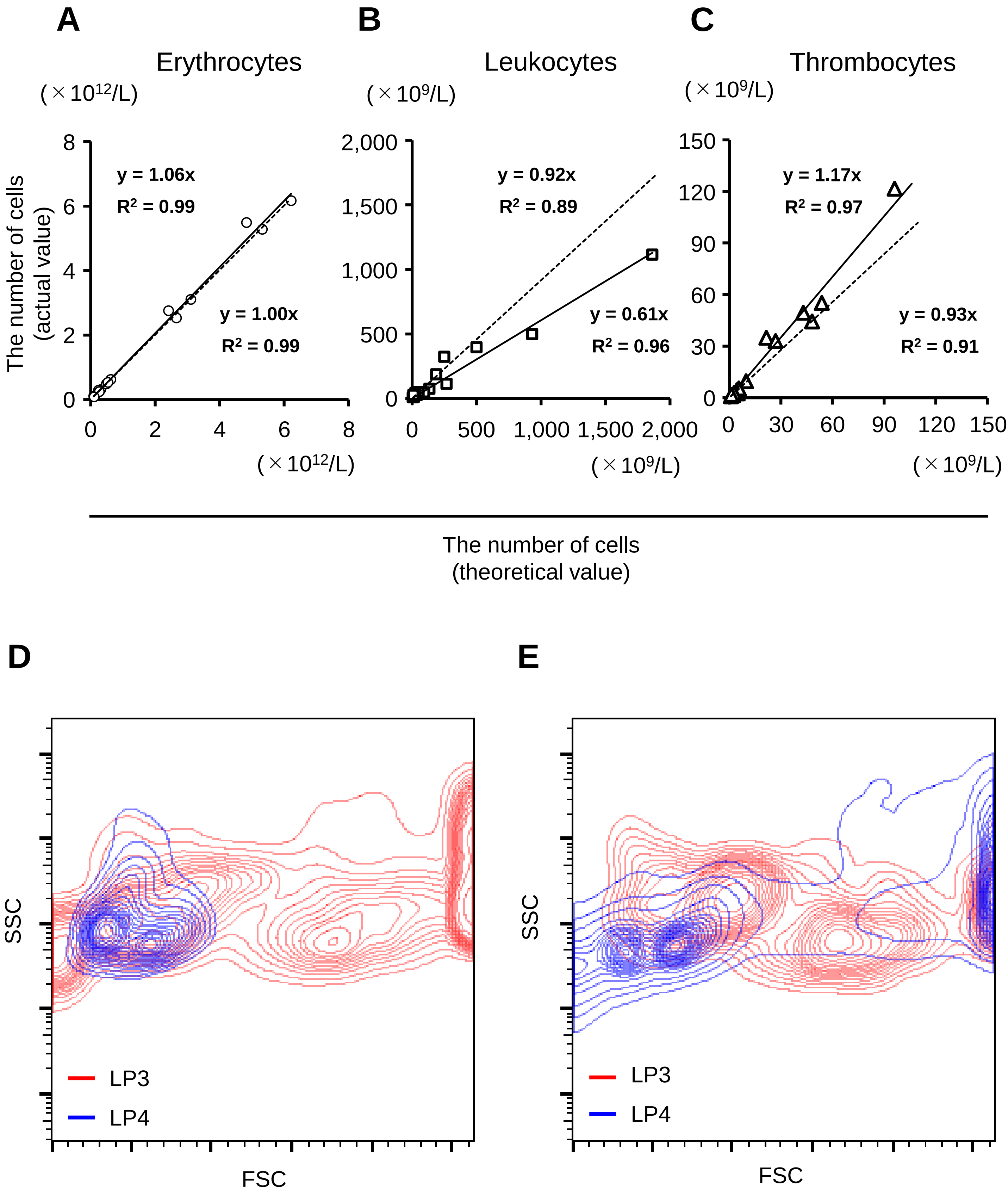

Supplementary Figure S3. Variation of cell number in diluted PB

A–C: Correlation between theoretical value and actual value in diluted PB.

The number of **A;** erythrocytes, **B;** leucocytes, and **C;** thrombocytes was identified. The number of leukocytes was determined using FCM. A dashed line indicates a regression line drawn with the data from 1:500 to 1:2,500, and a solid line indicates a regression line drawn with the data from 1:25 to 1:2,500. **D:** LP3 and LP4 were developed using FSC and SSC; blood cells stained at a ratio of 1:500. **E:** LP3 and LP4 were developed using FSC and SSC; blood cells stained at a ratio of 1:25.

Supplementary Figure S4

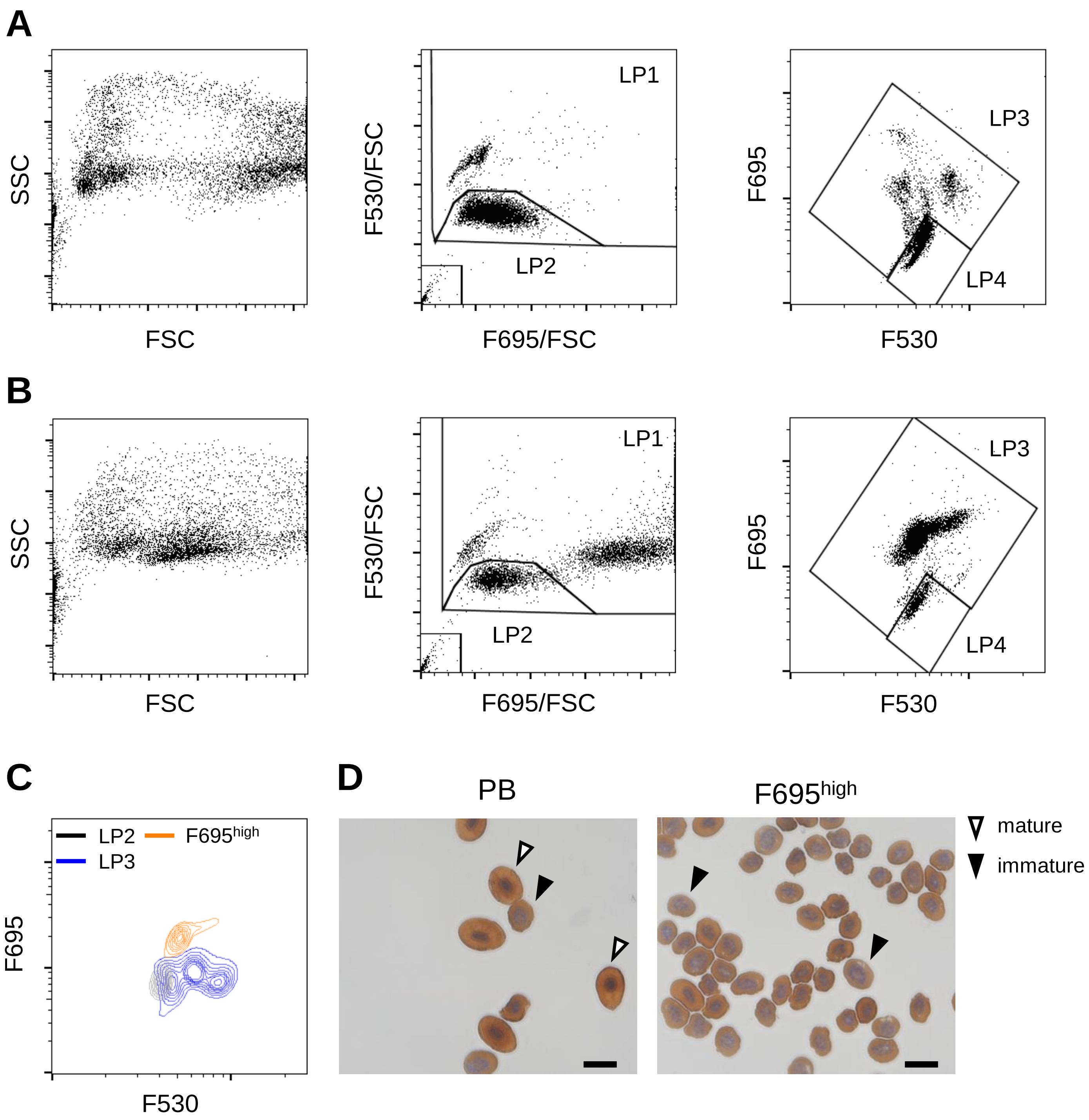

Supplementary Figure S4. Strategy of immature erythrocytes analysis using acridine orange
A: Scattergram analysis of control peripheral blood, analysed using forward-scattered light (FSC) and side-scattered light (SSC) (left panel), indicated cellular F695/FSC and cellular F530/FSC intensity (centre panel). The LP1 fraction was developed based on the F530 and F695 fluorescent intensity (right panel). **B:** Scattergram analysis of anaemic peripheral blood, analysed using FSC and SSC (left panel), as well as the F695/FSC and F530/FSC (centre panel). The LP1 fraction was developed based on the F530 and F695 fluorescent intensity (right panel). The high F695/FSC population (LP1) and high red fluorescent intensity population (LP3) were increased. **C:** LP2, LP3, and F695^{high} were developed based on the amount of F530 and F695 fluorescent intensity. F695^{high}: immature erythrocytes.
D: The peripheral blood (PB) and F695^{high} fractions were analysed by *o*-dianisidine staining. Cells in the F695^{high} fraction had a high N/C ratio and were weakly stained with *o*-dianisidine.

Supplementary Figure S5

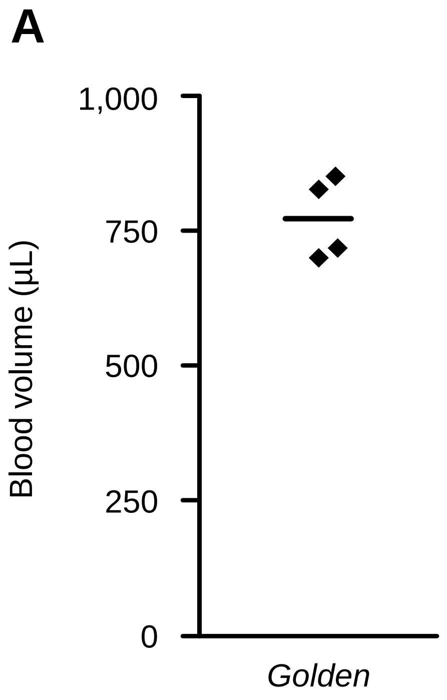

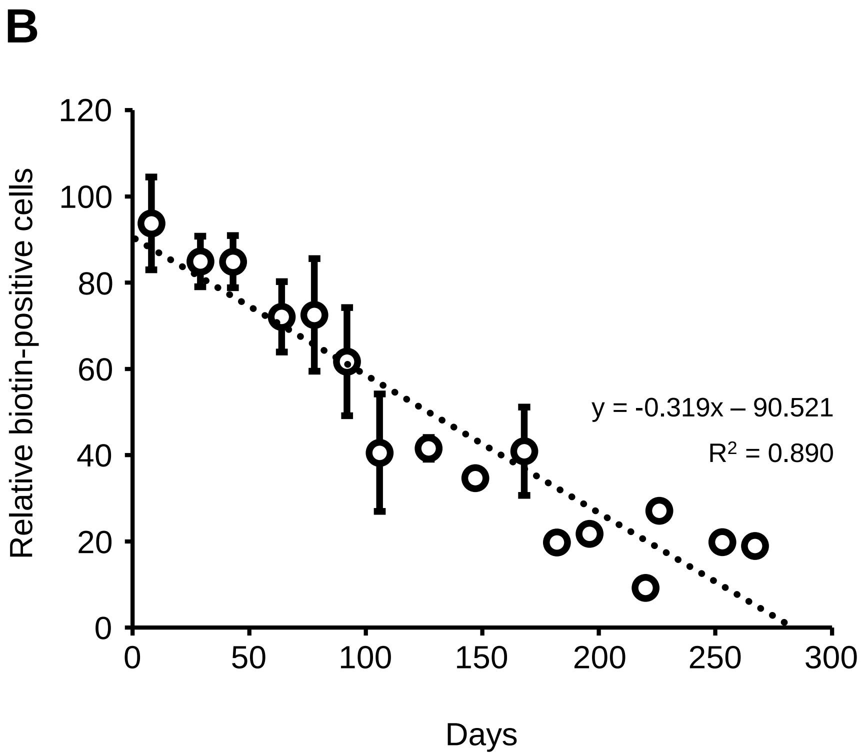

Supplemental Figure S5. Blood volume and erythrocyte lifespan in *Xenopus tropicalis*
A: Blood volume in *X. tropicalis* (*Golden* line). A Solid line indicates the mean blood volume value. **B:** Analysis of erythrocyte lifespan in *X. tropicalis* (*Golden* line) as the proportion of biotin-labelled cells. Day 8 and day 29: n = 6; day 43 and day 78: n = 5; day 64 and day 92: n=4; day 106, day 127, day 147, and day 182: n = 2; day 168, day 196, day 220, day 226, day 253, and day 267: n = 1.

Supplementary Table 1: Complete blood counts in *X. laevis* and *X. tropicalis*

		X. laevis		*X. tropicalis*					
		Wild type (n = 17)		*Golden* (n = 5)		*Nigerian H* (n = 6)		*Ivory Coast* (n = 6)	
	Fraction	Mean	SD	Mean	SD	Mean	SD	Mean	SD
Erythrocyte ($\times10^{12}$/L)	LP2 / TP2	0.87[b, d]	0.15	1.32[a, c]	0.13	0.87[b, d]	0.17	1.17	0.21
Granulocyte ($\times10^{9}$/L)	LP3 / TP3	31.84[b]	18.46	32.23[a, c]	6.89	15.82[b]	6.40	19.65	1.46
Lymphocyte ($\times10^{9}$/L)	LP5 / TP5	18.02[c]	17.86	29.59	28.05	57.82	45.80	36.48	25.67
Thrombocyte ($\times10^{9}$/L)	LP6 / TP6	11.53[b]	10.62	21.73[a, d]	2.68	17.89	15.32	6.81[b]	7.96
Haematocrit (%)		32.8[b]	6.28	43.9[a, c]	11.34	27.3[b]	7.53	33.8	2.69
Haemoglobin (g/dL)		7.8[b]	1.49	11.2[a, c]	2.10	6.9[b]	1.22	8.8	1.48
MCV (fL)		380.8[d]	54.10	330.6	74.27	312.1	40.16	295.0[a]	44.11
MCH (pg)		90.7[d]	9.59	84.3	10.2	81.2	11.1	76.8[a]	13.4
MCHC (g/dL)		24.2	3.78	26.6	6.69	26.4	5.47	26.2	4.40

[a, b, c, d] indicate the significance level of $p < 0.05$ vs. *X. laevis*, $p < 0.05$ vs. *Golden*, $p < 0.05$ vs. *Nigerian H*, and $p < 0.05$ vs. *Ivory coast*, respectively.
